# Peptide-conjugated phosphodiamidate oligomer-mediated exon skipping has benefits for cardiac function in *mdx* and *Cmah-/-mdx* mouse models of Duchenne muscular dystrophy

**DOI:** 10.1371/journal.pone.0198897

**Published:** 2018-06-18

**Authors:** Alison M. Blain, Elizabeth Greally, Graham McClorey, Raquel Manzano, Corinne A. Betts, Caroline Godfrey, Liz O’Donovan, Thibault Coursindel, Mike J. Gait, Matthew J. Wood, Guy A. MacGowan, Volker W. Straub

**Affiliations:** 1 Institute of Genetic Medicine, Newcastle University, International Centre for Life, Times Square, Newcastle upon Tyne, United Kingdom; 2 Department of Physiology Anatomy and Genetics, University of Oxford, Oxford, United Kingdom; 3 Medical Research Council, Laboratory of Molecular Biology, Cambridge, United Kingdom; 4 Department of Cardiology, Freeman Hospital, Newcastle upon Tyne, United Kingdom; University of Minnesota Medical Center, UNITED STATES

## Abstract

Cardiac failure is a major cause of mortality in patients with Duchenne muscular dystrophy (DMD). Antisense-mediated exon skipping has the ability to correct out-of-frame mutations in DMD to produce truncated but functional dystrophin. Traditional antisense approaches have however been limited by their poor uptake into cardiac muscle. The addition of cell-penetrating peptides to antisense molecules has increased their potency and improved their uptake into all muscles, including the heart. We have investigated the efficacy of the Peptide-conjugated phosphodiamidate morpholino oligomer (P-PMO) Pip6a-PMO, for restoration of cardiac dystrophin and functional rescue in DMD mice- the *mdx* mouse and the less well characterised *Cmah-/-mdx* mouse (which carry a human-like mutation in the mouse Cmah gene as well as a mutation in DMD). In our first study male mdx mice were administered Pip6a-PMO, i.v, fortnightly from 12 to 30 weeks of age alongside mock-injected age-matched mdx and *C57BL10* controls. Mice received 4 doses of 18 mg/kg followed by 8 doses of 12.5 mg/kg. The cardiac function of the mice was analysed 2 weeks after their final injection by MRI followed by conductance catheter and their muscles were harvested for dystrophin quantification. In the second study, male *Cmah-/-mdx* mice, received 12.5 mg/kg Pip6a-PMO, i.v fortnightly from 8 to 26 weeks and assessed by MRI at 3 time points (12, 18 and 28 weeks) alongside mock-injected age-matched mdx, *C57BL10* and *Cmah-/-mdx* controls. The mice also underwent MEMRI and conductance catheter at 28 weeks. This allowed us to characterise the cardiac phenotype of *Cmah-/-mdx* mice as well as assess the effects of P-PMO on cardiac function. Pip6a-PMO treatment resulted in significant restoration of dystrophin in mdx and *Cmah-/-mdx* mice (37.5% and 51.6%, respectively), which was sufficient to significantly improve cardiac function, ameliorating both right and left ventricular dysfunction. *Cmah-/-mdx* mice showed an abnormal response to dobutamine stress test and this was completely ameliorated by PIP6a-PMO treatment. These encouraging data suggest that total restoration of dystrophin may not be required to significantly improve cardiac outcome in DMD patients and that it may be realistic to expect functional improvements with modest levels of dystrophin restoration which may be very achievable in future clinical trials.

## Introduction

Duchenne muscular dystrophy (DMD) is a degenerative genetic muscle disease that occurs in around 1 in 3500 live male births [1]. The condition is caused by mutations in a large 79-exon gene encoding the 427 kDa cytoskeletal protein dystrophin, which forms a critical part of the dystrophin-associated glycoprotein complex (DGC) [2–3]. The role of the DGC is best characterised in muscle where it stabilises the muscle membrane and protects the muscle from contraction-induced damage. Loss of dystrophin from the muscle membrane results in sarcolemmal instability, dysregulation of calcium signalling and a cycle of events which culminates in fatty replacement of muscle and reduced strength. Although steroid treatment and nocturnal ventilation have increased the longevity of boys with DMD, patients continue to die from respiratory and cardiac complications in their 30s.

Patients with DMD commonly harbour out-of-frame or nonsense mutations which result in a complete loss of dystrophin [4]. However, evidence from patients with the milder allelic form of the disease, Becker muscular dystrophy (BMD), suggests that dystrophin can be partially functional, even with the presence of an internal deletion, if the “reading frame” is preserved [5–6]. The concept of exon skipping therapy for DMD (whereby a mutation-specific antisense oligonucleotide induces ‘skipping’ of the mutated exon during translation) was therefore conceived over a decade ago and remains one of the most rational therapeutic strategies under development [7].

One of the most extensively studied class of antisense oligonucleotide is the phosphodiamidate morpholino oligomer (PMO). The toxicological profile of long term PMO administration is favourable [8–9] and dystrophin restoration from a phase 2 clinical dose escalation study using PMO (AVI-4658) has been encouraging [10]. Indeed, Exondys 51 (eteplirsen), an antisense oligonucleotide indicated for the treatment of DMD patients amenable to exon 51 skipping, was granted accelerated approval by the U.S Food and Drug Administration in September 2016 (http://www.fda.gov/newsevents/newsroom/pressannouncements/ucm521263.html). However, little is known if PMO has any efficacy in patients’ hearts and indeed, what the long-term cardiac consequences could be. Early *in vivo* mouse studies using systemic PMO have consistently shown poor or absent dystrophin restoration in the heart [11–12]. A study in mice has also highlighted the potential danger of aggravating the cardiac phenotype when therapies are muscle-centric [13]. Furthermore, the death of DMD patients is usually due to cardiac failure. Therefore, it is exceptionally important to consider the efficacy of exon skipping in heart.

PMO conjugation to cell penetrating peptides has allowed heart dystrophin restoration to be significantly improved leading to functionally significant consequences [14–15]. More specifically, the Pip6 series of cell penetrating peptides (from the Gait laboratory, Cambridge University) have increased PMO exon skipping activity in heart by virtue of a hydrophobic core within the peptide [16].

Here we evaluate the efficacy of Pip6a-PMO in the well characterised *mdx* mouse (Study 1) which has a relatively mild phenotype. We also assess efficacy of PiP6a-PMO in a relatively new model of DMD, the *Cmah-/-mdx* mouse (Study 2), which has been reported to have a more severe phenotype with respect to cardiac and muscle pathology and function [17]. We also take the opportunity to more fully characterise the heart phenotype in this new mouse model compared to *mdx* mice.

## Methods

### Animals

Male *mdx* from Jackson laboratories (Bar Harbor, ME, USA) (n = 15 P-PMO injected, n = 9 veh.) and male *C57BL10* mice (Jackson laboratories) (n = 11, saline vehicle (veh.)) were used in the first investigation. Mice were 12 weeks old at the beginning of the study, received their final injection at 30 weeks old and were analysed 2 weeks later at 32 weeks old.

In the second study male *Cmah-/-mdx* mice (n = 9 P-PMO injected, n = 13 veh.) which were bred from heterozygous mice obtained from Jackson Laboratories (catalogue number 017929) were analysed alongside male *C57BL10* (n = 12 veh.) and *mdx* (n = 9 veh.) mice. Mice were 8 weeks old (younger than for the first study as a more severe phenotype was expected in the *Cmah-/-md*x mice) at the beginning of the study, received their final injection at 26 weeks old and were analysed at 12, 18 and 28 weeks.

Mice were housed under controlled temperature (17–28°C) and light conditions (12:12h light:dark cycle). Animals had free access to food and water. Mice were cared for and received daily welfare checks by the animal house technicians as well as additional checks by the researcher for adverse clinical signs, directly after and periodically for several hours after an intervention. The investigations conformed with the Guide for the Care and Use of Laboratory Animals published by the US National Institutes of Health [NIH Publication No.85-23, revised in 1985 and was performed under the terms of the Animals (Scientific Procedures) Act 1986, authorized by the Home Secretary, Home Office UK]. All experiments were performed at the animal care facility of Newcastle University, UK. The study was approved by the Ethical Review Committee of Newcastle University.

### P-PMO synthesis

Pip6a-PMO was synthesised in the Gait laboratory, MRC Laboratory of Molecular Biology in Cambridge. This peptide-PMO was conjugated through a stable amide linkage using methods described previously [16]. Briefly, PMO (PMOME23, sequence GGCCAAACCTCGGCTTACCTGAAAT (from GeneTools)) was conjugated through its secondary amine (morpholine) to the C terminus of a peptide synthesized in the laboratory. Peptide-PMO was then purified and desalted on a column. The work described in this paper formed part of a large collaborative project conducted by the MDEX consortium. The decision to use PIP6a-PMO for these studies was made based on pilot studies conducted in the Wood lab which suggested this was the most efficacious P-PMO (both *in vivo and in vitro*) available to us at the time of this study.

### Delivery of P-PMO

A summary of the timelines for treatment and analysis of mice in Study 1 and Study 2 can be seen in S1 Fig. Following induction of anaesthesia with 5% isofluorane in an anaesthetic chamber, mice were transferred to a mask and maintained at 1.5% isofluorane for the duration of the injection (5–10 mins). An infra-red lamp was used to warm the tail and dilate the tail veins and the P-PMO was injected slowly i.v. using an insulin syringe (Terumo). For the second study in *Cmah-/-mdx* mice, a tail vein cannula connected to a saline syringe which was then switched to the P-PMO syringe was used as a refinement of the delivery technique. Mice were placed in cages in an incubator and observed for adverse clinical signs until recovered fully (10–30 mins). Mice were returned to the home cage with their cage mates only after they were fully recovered. Mice received 4 fortnightly injections of 18 mg/kg (the initial dose was chosen based on the maximum dose feasible based on P-PMO availability and also preliminary studies in the Wood lab (which showed dystrophin restoration with a single dose of 18 mg/kg P-PMO). The dose was then adjusted because of mortality in the mice and the remaining mice received 6 fortnightly injections of 12.5 mg/kg (in an injection volume of 160 μl) (a dose used in previous studies in the Wood lab). In the second study, mice received fortnightly injections of 12.5 mg/kg. Vehicle injected controls underwent the same anaesthesia protocol as P-PMO-injected animals and were injected with 160 μl 0.9% saline. The rationale for delivering P-PMO at fortnightly intervals was based on studies in the Wood lab which showed that dystrophin could be observed for at least two weeks following a single injection of P-PMO. At the time of this study, there had been no studies of pharmacokinetics or biodistribution of Pip6a-PMO, although an ELISA based method is now available [18].

### Right and left ventricular functional assessment by cardiac MRI

Following induction of anaesthesia with 5% isofluorane in an anaesthetic chamber, the tail vein was cannulated and mice were laid prone on a cradle (Dazai Research Instruments, MICe, Toronto, Canada) that allowed monitoring of body temperature, respiratory rate and heart rate. Mice were placed in the magnet and anaesthesia was maintained using 1–1.8% isofluorane via a nose cone. Body temperature was maintained using a warm air blower. Images were acquired on a 7 Tesla horizontal bore Varian microimaging system equipped with a 12-cm microimaging gradient insert (maximum gradient 40 gauss/cm) (Varian Inc., Palo Alto, CA, USA). Mice were scanned in a 39mm diameter quadrature birdcage volume coil (Rapid Biomedical GmbH, Wurzburg, Germany). Following power calibration and global shimming a series of four pilot transverse images were acquired over the heart. Single slice coronal and sagittal images were then obtained in order to view the apex and mitral valve planes. These images were used to plan for the true short axis plane. To measure ventricular function, twelve contiguous short axis slices were acquired to cover the entire left and right ventricles using a spoiled gradient-echo cine sequence (TR = 5ms, TE = 1.42ms, flip angle 15°, FOV 30x30mm, data matrix 128x128, 1mm slice thickness). Images were ECG triggered to the R wave with a cine delay of 15ms and typically 30 phases were acquired distributed through the cardiac cycle. Images were zero-filled to a matrix size of 256x256. Scans were converted to matfiles using a matlab script (kindly provided by Dr Johannes Riegler, UCL) and analysed using the freely available analysis software Segment v1.8 (http://segment.heiberg.se) to give LV and RV functional parameters.

### Assessment of myocardial calcium influx by manganese enhanced MRI (MEMRI)

60 mM manganese chloride (Sigma-Aldrich 244589) was given by intravenous infusion through the tail vein cannula at a flow-rate of 0.6ml/hour [19–20]. Flow time was adjusted according to weight to give a total dose of 190 nmol/g body weight (for example, for a 30g mouse, this would result in a 9.5 minute infusion). Gradient echo short axis images at the level of the papillary muscles (T_1_ weighted parameters: TR = 35 ms, TE = 3.5 ms, flip angle 60°, FOV 30 mm x 30 mm, data matrix 128 x 128, 1mm slice thickness, 6 averages) were taken. Prior to manganese infusion four baseline images were acquired in order to average any variations due to changes in TR as a result of fluctuations in heart rate. Infusion was begun and images were taken at 2.5 mins, 5 mins and then at 5 minute intervals thereafter, for 30 minutes. A relative increase in T_1_ weighted contrast indicates increased manganese uptake. Images acquired from each time point were opened in Image J (http://rsb.info.nih.gov/ij/) and converted to a stack using the stack builder plugin. An area of interest was drawn to fit inside the myocardium and average signal intensity was measured. If necessary, minor adjustments of this drawn region were made for subsequent images in the stack and the increase in myocardial contrast enhancement was expressed as a percentage increase from the average of the four baseline images (which showed little or no variation). MEMRI data is expressed as an enhancement ratio over baseline.

### Cardiac conductance catheter

Measurements were made in the same mice which had previously undergone MRI assessment however data was obtained from only a subset of animals because of the technical difficulty of the conductance catheter technique (see figure legends for group sizes). Conductance catheter measurements were made 1–3 days following MRI in closed-chest, spontaneously breathing mice anaesthetised by isofluorane. Following induction of anaesthesia in a chamber using 5% isofluorane, animals were transferred to a nose cone in a supine position and maintained at around 2% isofluorane in oxygen. Body temperature was maintained at 37°C using a homoeothermic blanket (Harvard apparatus). A 1.4-French conductance catheter (Millar) was introduced into the right carotid artery and advanced retrogradely across the aortic valve into the left ventricle. The catheter was advanced under continuous haemodynamic monitoring to ensure proper placement in the left ventricle. After stabilization, steady-state measurements were recorded.

A small laparotomy was made at the level of the xiphisternum and transient compression on the inferior *vena cava* was applied using a flexible instrument cap to reduce preload used in determining end-systolic elastance (E_es_) (a preload independent measure of contractility). Measurements were taken under steady state, inferior vena cava occlusion and in the second study in *Cmah-/-mdx* mice, during infusion of dobutamine (5 μg/kg/min). Measurements were also taken during infusion of 10 μg/kg/min dobutamine however a number of the mutant mice became unstable and therefore the resultant data have not been included in this manuscript. Volume was calculated using the Relative Volume Units/Cuvette method in which external blood-filled standards of known volume are used to calculate a slope and intercept to convert the conductance catheter signal of relative volume units to volume. This blood conductance signal needs to be corrected for the parallel conductance attributed to the tissues surrounding the left ventricular cavity. Parallel conductance (V_p_) was estimated by the previously described hypersaline method in which a bolus of 10 μL of 10% hypersaline was injected through the left internal jugular vein [21]. The animals were euthanised after the experiment by cervical dislocation and their hearts excised, washed and weighed. In the second study, hearts were injected through the left ventricle with 1M KCl solution while still beating *in situ* (as a refinement to the dissection technique). This allowed the hearts to be preserved in diastole. In the first study, Tibialis anterior (TA) and diaphragm (DIA) muscles were also removed to allow a more comprehensive assessment of dystrophin restoration. Muscles were snap frozen in isopentane cooled over dry ice and stored at -80°C.

### qRT-PCR and RT-PCR quantification of *Dmd* exon 23 skipping

qRT-PCR: RNA was extracted from muscle sections using Trizol. One microgram of RNA was reverse transcribed using the High Capacity cDNA RT Kit (Applied Biosystems, Warrington, UK) according to manufacturer's instructions. qPCR analysis was performed using 25 ng cDNA template and amplified with Taqman Gene Expression Master Mix (Applied Biosystems, Warrington, UK) on a StepOne Plus Thermocycler (Applied Biosystems, Warrington, UK). Levels of *Dmd* exon 23 skipping were determined by multiplex qPCR of FAM-labelled primers spanning Exon 20–21 (Assay Mm.PT.47.9564450, Integrated DNA Technologies, Leuven, Belgium) and HEX-labelled primers spanning Exon 23–24 (Mm.PT.47.7668824, Integrated DNA Technologies, Leuven, Belgium). The percentage of *Dmd* transcripts containing exon 23 was determined by normalizing exon 23–24 amplification levels to exon 20–21 levels.

RT-PCR: Four hundred nanograms of RNA template was used in a 50 μl reverse transcription reaction using One Step RT-PCR Kit (Qiagen, Hilden, Germany) and gene-specific primers (Ex 20–26, Fwd: 5′-CAG AAT TCT GCC AAT TGC TGA G-3′, Rev: 5′-TTC TTC AGC TTG TGT CAT CC-3′). Cycle conditions: 50°C for 30 minutes, followed by 30 cycles of 30 seconds at 94°C, 1 minute at 58°C, and 2 minutes at 72°C. Two microliters of cDNA was further amplified in a 50 μl nested PCR (QIAGEN PCR kit) using the following cycle conditions: 94°C for 30 seconds, 58°C for 1 minute, and 72°C for 1 minute for 24 cycles (Ex 20–26: Fwd: CCC AGT CTA CCA CCC TAT CAG AGC, Rev: CCT GCC TTT AAG GCT TCC TT). PCR products were examined by electrophoresis on a 2% agarose gel.

### Western blot quantification of dystrophin restoration

Briefly, 90x 7 μm transverse cryosections lysed in 300 μl buffer (75mM Tris-HCl pH 6.8, 10% SDS, 5% β-Mercaptoethanol and protease/phosphatase inhibitors, Roche) and homogeneized in a Precellys 24 (Peqlab) prior to centrifugation at 5000 rpm (Heraeus, #3325B) for 3 min and boiling. Protein was quantified by Bradford assay and 3 μg protein/sample were resolved in a NuPage a 3–8% Tris–Acetate gel (Invitrogen). Proteins were transferred to a 0.45 μm pore size PVDF membrane for 140 min at 30V and membrane blocked with Odyssey buffer for 1 hour. Blocked membranes were probed O.N. at 4°C with primary monoclonal anti-dystrophin (1:200, NCL-DYS1, Novocastra) and anti-vinculin (loading control, 1:100 000, hVIN-1, Sigma) antibodies. Secondary antibody IRDye 800CW goat anti-mouse was used at a dilution of 1:20000 (LiCOR). Fluorescent signals for both proteins were recorded and quantified using the Odyssey imaging system. The levels of dystrophin expression were normalized to vinculin, and P-PMO treated samples were referred to 10 and 5% C57BL/10.

### Histology

Muscles were mounted onto cork with OCT and 8 μm fresh frozen sections were cut and collected onto poly-sine slides (VWR). In the case of the heart, the sections were cut transversely at the level of the papillary muscles for the first study. In the second study sections were taken at 6 different levels. For the TA and QUAD, transverse sections were collected approximately mid-way along the muscle. Hemi-diaphragm pieces were rolled and mounted such that transverse sections could be cut though the full width.

### H&E staining

Sections were stained using a standard H&E protocol. The investigator was blinded to treatment and tissue sections were examined for the presence of central nucleation, fibrosis, necrosis, inflammatory infiltration and changes in fibre diameter and given a subjective ‘severity score’ to guide further analysis. In the case of the heart, changes to the structure of the ventricle and thickness of the ventricular walls were noted.

### Masson’s Trichrome staining

Severity scores on H&E stained heart sections from Study 2 suggested a treatment effect on pathology and we were interested to learn if fibrosis differed between the *mdx* and *Cmah-/-mdx* models. Heart sections were stained using the Accustain Massons Trichrome histology kit (Sigma Aldrich) according to manufacturer’s instructions. Whole heart cross sections were captured using a 1.25x objective. Areas of fibrosis were delineated using the wand tool in ImageJ on 6 sections/animal. Fibrosis was calculated as a percentage of the total cross sectional area. One mouse in the *Cmah-/-mdx* untreated group showed a particularly high level of fibrosis (32%) and was identified as a significant outlier by Grubbs testing and removed from the analysis.

### Immunohistochemistry

A double staining protocol for laminin and dystrophin was followed. Briefly, air dried slides were soaked in PBS and then blocked for 30 mins with goat serum (Abcam ab7481) in PBS. Primary antibodies (dystrophin anti-rabbit (Abcam ab15277) and Laminin alpha-2 chain anti-rat (Sigma L0663)) were used at a concentration of 1:500–1:1000 and applied for 30–60 mins at room temperature. Secondary antibodies (Alexa Fluor 594 goat anti-rabbit and Alexa fluor 488 goat anti-rat (Invitrogen) were used at a concentration of 1:1000 and applied for 30 mins-2 hr protected from light and at room temperature. Coverslips were mounted using ProLong Gold Antifade reagent. In the first study, sarcolemmal γ-sarcoglycan expression was also evaluated in heart and TA sections Anti-GSGC Rabbit polyclonal, Proteintech Europe #18102-1-APm (used 1:200)). nNOS expression was evaluated in TA (nNOS Rabbit Polyclonal Antibody, Life Technologies #617000 (used 1:400)). Sections were incubated overnight at 4°C in a single primary antibody (in 4% BSA in PBS). Secondary antibody (Alexa Fluor 594 anti-rabbit (Invitrogen)) was used at a concentration of 1:1000 and applied for 30mins-2hours.

### Microscopy and dystrophin quantification

5 images were acquired for each muscle using a Zeiss microscope with x20 objective. Exposure settings were set to a constant using the *C57BL10* control slide which had the highest level of fluorescence. Apart from identification of the *C57BL10* control slides for adjustment of exposure settings during microscopy, the analyst was blinded to the treatment which the dystrophic mice had received. Images were imported into Volocity software (PerkinElmer) and from each image ten random 30x30 pixel regions of interest (ROI) were created on the muscle membrane using the laminin protein (control protein). The Dystrophin was then revealed and minor adjustments to the ROIs were made if necessary (to avoid areas of staining artefact etc.). Signal intensity from both channels was measured for all 50 ROIs and exported to an excel spreadsheet, normalised to laminin signal and expressed as a % dystrophin recovery score. To account for background staining due to technique and naturally occurring revertant fibres, recovery was scaled against *mdx* and *Cmah-/-mdx* intensities which were set to give an average of 0% recovery. At least two *C57BL10* control slides were used to give an average value for 100% recovery.

### Quantification of centralised nuclei and fibre size

Centrally nucleated and non-centrally nucleated fibres were counted on 4 random fields of view (10x magnification) on H&E stained sections using the ‘cell counter’ plugin in Image J. % centralised nuclei was calculated from number of centrally nucleated cells divided by total number of cells counted.

Fibre size was measured using minimal Feret’s diameter on Image J. 4 laminin stained images (x20 magnification) per animal were analysed. Images were opened in Image J, adjusted so that the brightness was optimal and then converted to 8 bit. The image was then thresholded and the ‘analyse particles’ function was used to measure the minimal Feret’s diameter [22]

### Statistics

Differences in individual functional parameters between groups were assessed by 1-way ANOVA. Where a significant (p≤0.05) main effect was identified, post-hoc LSD (1^st^ study) or Bonferroni tests (2^nd^ Study) were used to identify where the differences lay. For the histological data, treatment effect was analysed using unpaired Student’s t-tests with significance set at p≤0.05. Effect of dobutamine stress on each group of mice was analysed using paired t-tests with significance set at p≤0.05.

## Results

### Effect of Pip6a-PMO on animal health and mortality

There were some deaths (5 out of the 15 mice that started the treatment) in the Pip6a- P-PMO treated group of mice in Study 1. These deaths generally occurred immediately after injection and after 2 (one mouse) or 3 (4 mice) repeat injections, at the higher dose of 18 mg/kg, suggesting a possible cumulative toxic effect of the higher treatment dose. The sudden nature of the deaths is perhaps suggestive of a shock syndrome which would be difficult to confirm through histological examination. Nevertheless, histological examination of the hearts of the mice showed that one mouse had dilated cardiomyopathy however this is not an unusual observation for this mouse strain. Macroscopic examination of the major organs *post mortem* did not reveal any obvious cause of death. No deaths were seen in the mock injected mice suggesting that the deaths in the P-PMO mice were not due to repeated anaesthesia or volume overload. One further mouse died during tail vein cannulation in preparation for MRI (two weeks post final injection). In Study 1 ten mice did however survive the full course of injections and could be assessed functionally. Indeed, if we consider animal body weight as a crude indicator of health status, there was no difference in body weight between the untreated and treated *mdx* (S1 Table). In Study 2, there were no deaths and treatment caused no change in body weight although *Cmah-/-mdx* mice were significantly smaller than *C57BL10* mice at baseline (S2 Table).

### P-PMO gives rise to exon skipping and dystrophin restoration in heart

Exon 23 skip could be detected in all P-PMO treated hearts from Study 1. An average of 18% of DMD transcripts lacked exon 23 but level of skip reached as high as 34% in the heart of one mouse.

**Fig 1:**
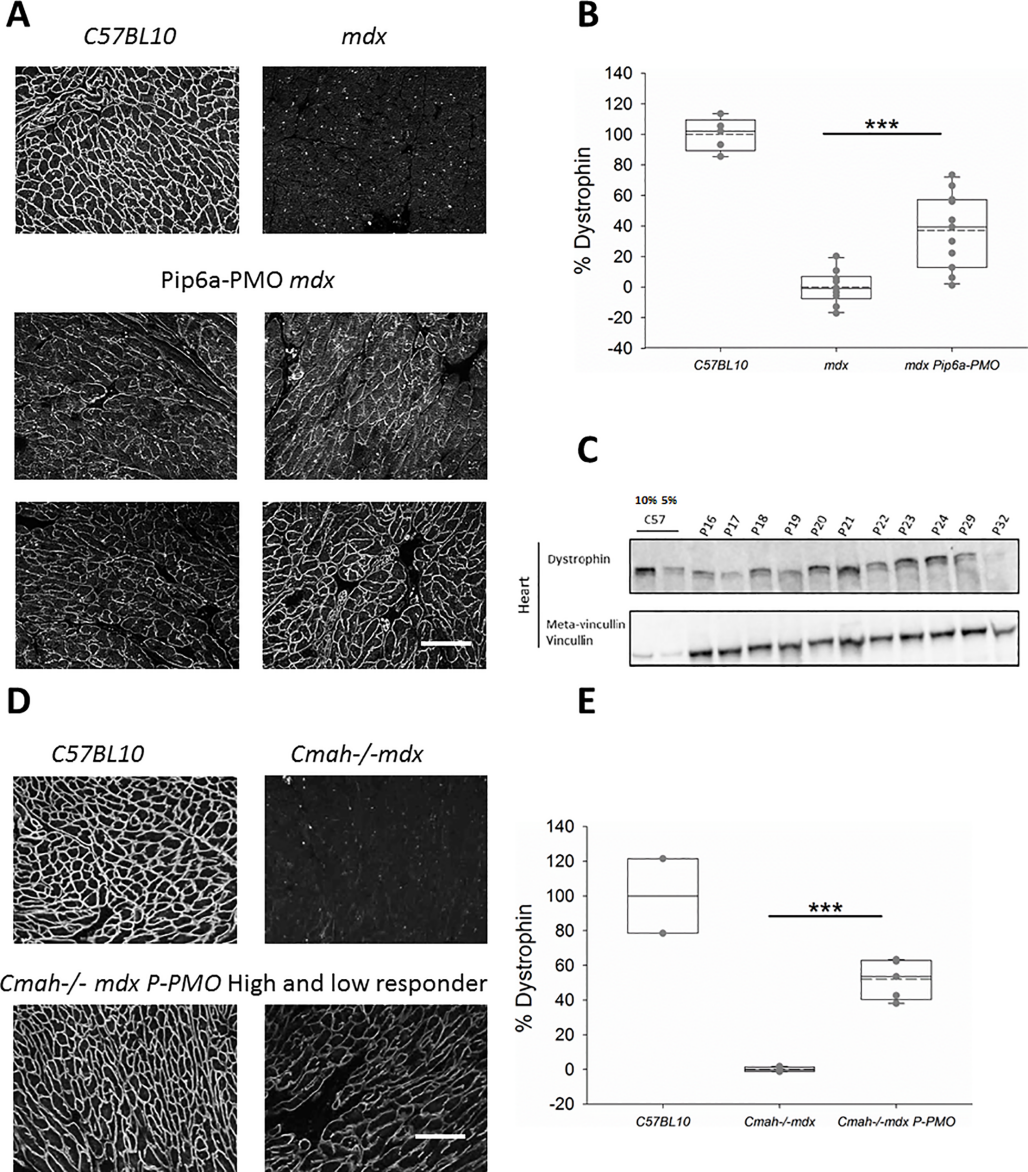
**A-E: Pip6a-PMO restores cardiac dystrophin in *mdx* and *Cmah-/-mdx* mice**. A) Immunofluorescent (IF) staining of dystrophin in *C57BL10*, control *mdx* and four P-PMO treated *mdx* mice demonstrating the homogeneity and range of restoration. B) Box plots showing quantification of dystrophin restoration on dystrophin/laminin IF. Average dystrophin restoration was 37.2% (***p<0.001, Unpaired Student’s t-test) (*C57BL10* n = 6, *mdx* n = 8 and *P-PMO treated mdx* n = 11 mice). C) western blot of cardiac dystrophin expression and meta-vinculin control in individual P-PMO treated *mdx* mice and *C57BL10* mice (10% and 5%), again demonstrating the range of restoration. *mdx* control samples which showed no band for dystrophin were ran in parallel on a separate gel. D) IF staining of dystrophin in *C57BL10*, control *Cmah-/-mdx* and examples of a high and low ‘responder’ (mice showing the best and worst levels of dystrophin restoration, respectively). E) Box plots showing quantification of dystrophin restoration on dystrophin/laminin IF. Average dystrophin restoration was 51.6%. (***p<0.001, Unpaired Student’s t-test). Scale bars = 100 μm (*C57BL10* n = 2, *Cmah-/-mdx* n = 6 and P-PMO treated *Cmah-/-mdx* 6 mice/group).

Pip6a-PMO successfully restored low levels of dystrophin fairly homogeneously throughout the right and left ventricle of *mdx* mice(Study 1) (Fig 1A) and *Cmah-/-mdx* mice (study 2) (Fig 1D). Indeed, in the sections examined, dystrophin was restored in 100% of cardiomyocytes. The level of restoration in the first study was 37.2% of levels in the WT animals (Fig 1B) and dystrophin levels reached as high as 66.4% (animal number p29) of WT levels by immunofluorescence.

Western blot (WB) in heart protein lysates confirmed good dystrophin restoration in *mdx* Pip6a-treated animals (Fig 1C) and showed similar variability in the level of response between animals. Animals p20 and p21 were identified as the best responders (animals showing highest levels of dystrophin restoration) using this method and these two animals were among the top three responders (mice showing the highest levels of dystrophin restoration) by immunofluorescence. Dystrophin restoration quantified by immunofluorescence also showed a moderate positive correlation with % exon skip (p = 0.014 (1-sided) Pearson Correlation) S2 Fig. Immunofluorescence quantification of dystrophin restoration is already validated and used in human trials and therefore this technique was chosen for Study 2.

Similarly, homogeneous levels of dystrophin restoration (with 100% of cardiomyocytes expressing dystrophin in the sections examined) were achieved in Study 2 with an average restoration of 51.6% of WT levels in the *Cmah-/-mdx* mice and levels up to 66.2% (Fig 1D & 1E).

### Effect of Pip6a-PMO on cardiac histological features

**Fig 2:**
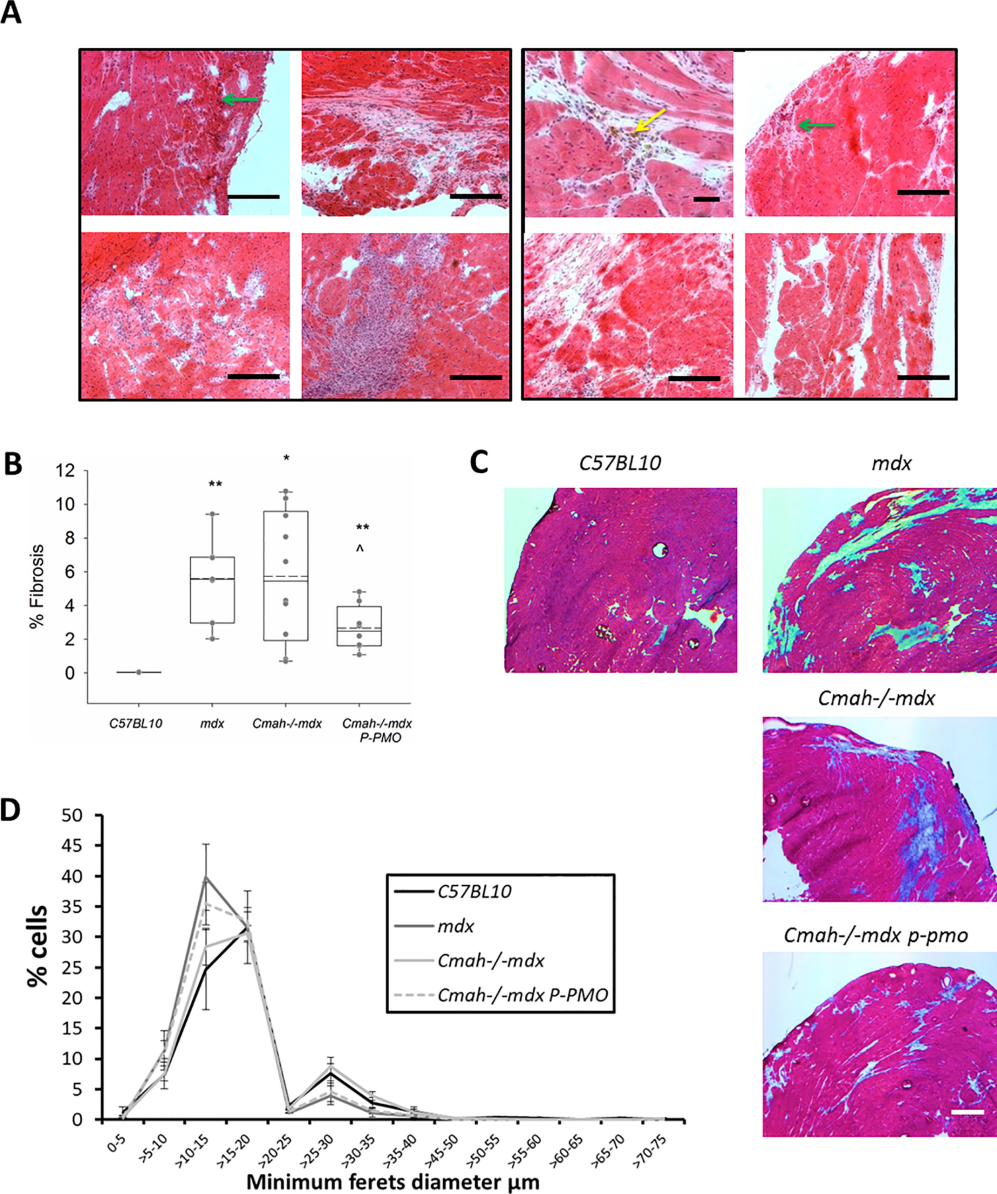
**A-D: P-PMO reduces fibrosis in early treated *Cmah-/-mdx* mice.** A) Treated and untreated *mdx* mice show a similar range of cardiac pathology but treated animals showed a distinct aspect of pathology- brown staining cells (yellow arrow) which are assumed to be cells rich in intrinsic pigment (such as melanin). H&E staining of heart demonstrating presence of fibrosis, necrotic fibres (green arrows) and (x10) Scale bar = 50 μm. B) Quantification of fibrosis on masson’s trichrome stained *Cmah-/-mdx* heart sections (*p<0.05, **p<0.01 different from *C57BL10*, ^P<0.05 different from *Cmah-/-mdx*, Unpaired Student’s t-tests) (n = 6-9/group) C) Masson’s trichrome stained sections of the most fibrotic animals in each group (Scale bar = 500 μm). D) P-PMO treatment does not significantly affect fibre size distribution (n = 6-9/group, 2-way ANOVA. Error bars represent SEM).

In Study 1 cardiac pathology varied quite widely between control *mdx* mice but in general, hearts showed widespread small patches of fibrosis across both ventricles (Fig 2A). Necrosis was present in a subset of hearts and the severity of this varied from a few fibres to a very large patch.

A similar range of pathology was also seen in the P-PMO treated animals (Fig 2A) although treated animals showed brown staining cells/deposits (assumed to be cells rich in melanin such as melanomacrophage) could only be seen in the treated animals.

In Study 2 *Cmah-/-mdx* mice showed greater variability in fibrotic content than *mdx* mice and contrary to previously published data [17], there were no significant differences in the levels of fibrosis between these two DMD models. It should however be noted that one *Cmah-/-mdx* mouse was identified as an outlier with 32% fibrosis and removed from the overall analysis. P-PMO significantly reduced fibrotic content of *Cmah-/-mdx* hearts (Fig 2B and 2C). Necrosis was not a feature in the *Cmah-/-mdx* mice (both treated and untreated) or the *mdx* mice at this age (28 weeks).

There was no significant difference in animal strains with regards to average fibre diameter and all mice showed a similar distribution of fibre sizes (Fig 2D). There was however a reduction in larger diameter cells between (30–50μm) in *Cmah-/-mdx* with treatment (p<0.05).

### Exon skip and dystrophin restoration in tibialis anterior

Exon skipping and dystrophin restoration in the TA and diaphragm of Pip6a-PMO treated *mdx* mice (Study 1) was also quantified to examine if those mice with highest dystrophin restoration in the heart were also ‘high responders’ in other muscles. High levels of exon 23 skip (50–70%) was detected in all TAs of P-PMO treated *mdx* mice (Study 1). Immunohistochemical quantification of dystrophin restoration in treated *mdx* TA was 96.4% of *C57BL10* levels (S3 Fig). Even animals which showed a low level of restoration in the heart showed excellent restoration in the TA muscle and in general, TA dystrophin restoration showed less variability between animals. Results from western blot mirrored those from immunohistochemistry with a higher level of dystrophin restoration in TA compared to heart (around 50%).

### Exon skip and dystrophin restoration in diaphragm

Exon 23 skip as measured by PCR was 35% in diaphragm. P-PMO caused a significant restoration of dystrophin such that dystrophin levels were 47.0% of *C57BL10* control in the treated animals (similar to the level achieved in the heart) (S3 Fig). Restoration in diaphragm was more variable than in TA and showed a moderate positive correlation to heart dystrophin restoration (R^2^ = 0.407). In the untreated *mdx*, large numbers of small cells (presumably, mast cells), which showed unspecific staining for dystrophin, could be seen. There is a marked reduction in these cells in the treated animals (S3 Fig).

### Effect of Pip6a-PMO on histological features in TA and DIA

32 week old *mdx* mice had relatively mild pathology in the TA muscle with little or no fibrosis but with around 80% centrally located nuclei (CLN) (S4 Fig). Treatment caused no significant reduction in CLN in the TA. There was however a significant effect of treatment on fibre size with P-PMO treated mice having significantly larger average fibre size (Feret’s min diameter) than untreated mice (p<0.001, highly significant) (S4 Fig). This was due to an increase in fibre size homogeneity and a reduced preponderance of small diameter fibres (S4 Fig). There was also a marked effect of treatment on the *mdx* diaphragm with significantly less pathology present in the treated animals (S4 Fig).

### Effect of dystrophin restoration on nNOS and γ-dystroglycan expression

γ- sarcoglycan was reduced or absent in untreated *mdx* TA and heart. nNOS staining was absent in *mdx* TA. P-PMO treatment restored both nNOS and γ-sarcoglycan to the sarcolemma (S5 Fig).

### P-PMO restores normal cardiac output in *mdx* mice

**Fig 3:**
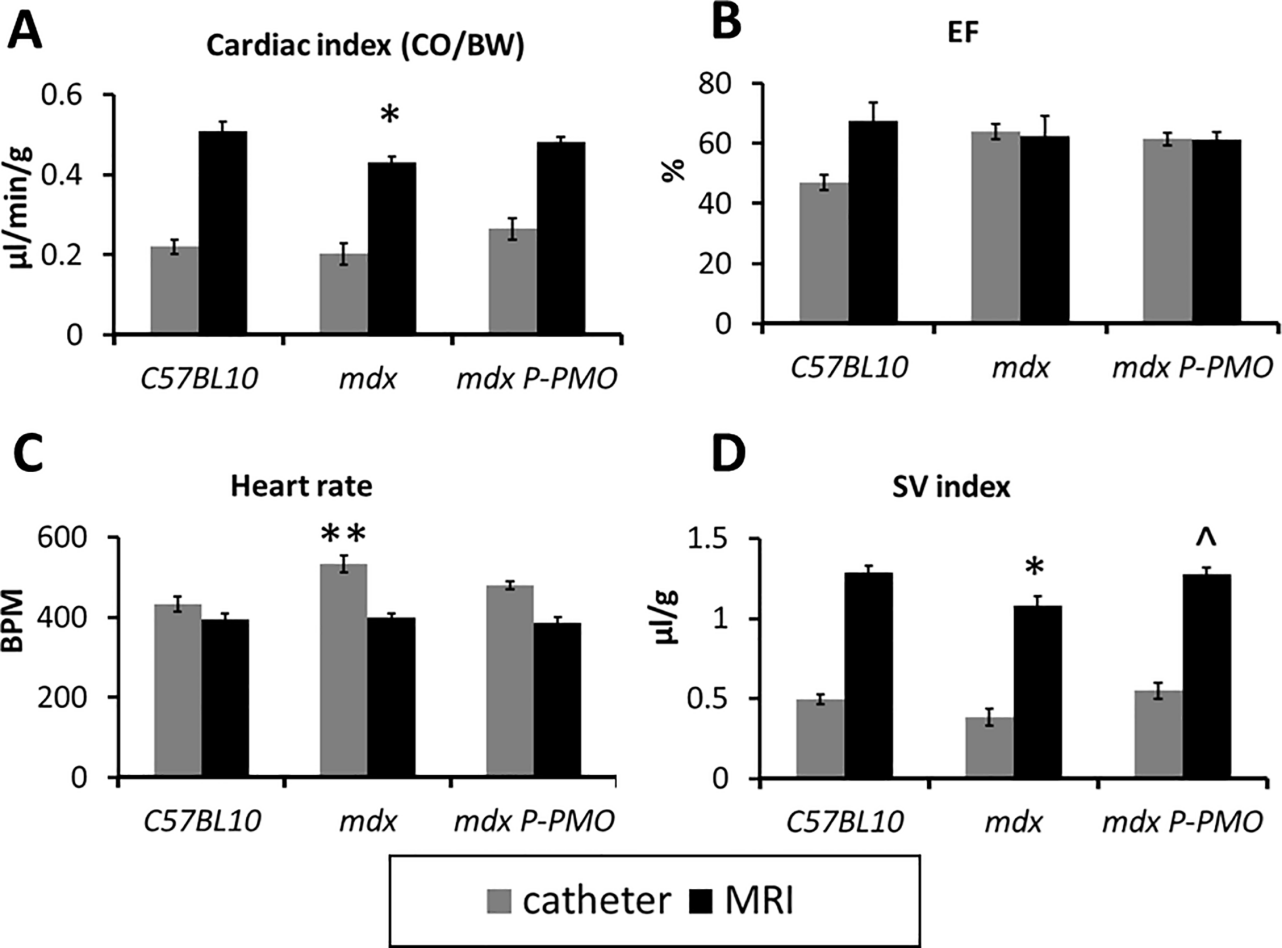
**A-D: P-PMO normalises cardiac output in *mdx* mice with increased stroke volume.** MRI (n = 9-11/group) and conductance catheter (n = 8-10/group) data is presented showing A) Cardiac output (indexed to body weight) B) ejection fraction C) heart rate and D) Stroke Volume index. (*p<0.05, **p<0.01 significantly different from *C57BL10*, ^P<0.05 significantly different from mdx, 1-way ANOVA, post-hoc LSD. Error bars represent SEM).

32 week old *mdx* mice have evidence of cardiomyopathy with reduced stroke volume and cardiac output (Fig 3A) despite preserved ejection fraction (Fig 3B). Pip6a-PMO restores normal cardiac output by virtue of increased stroke volume (Fig 3D). From the conductance catheter data, it is evident that the *mdx* mice attempt to compensate for reduced cardiac output by increased heart rate (Fig 3C). Pip6a-PMO treatment is accompanied by a significant reduction in heart rate (Fig 3C).

### P-PMO restores LV ejection fraction in *Cmah-/-mdx* mice

**Fig 4:**
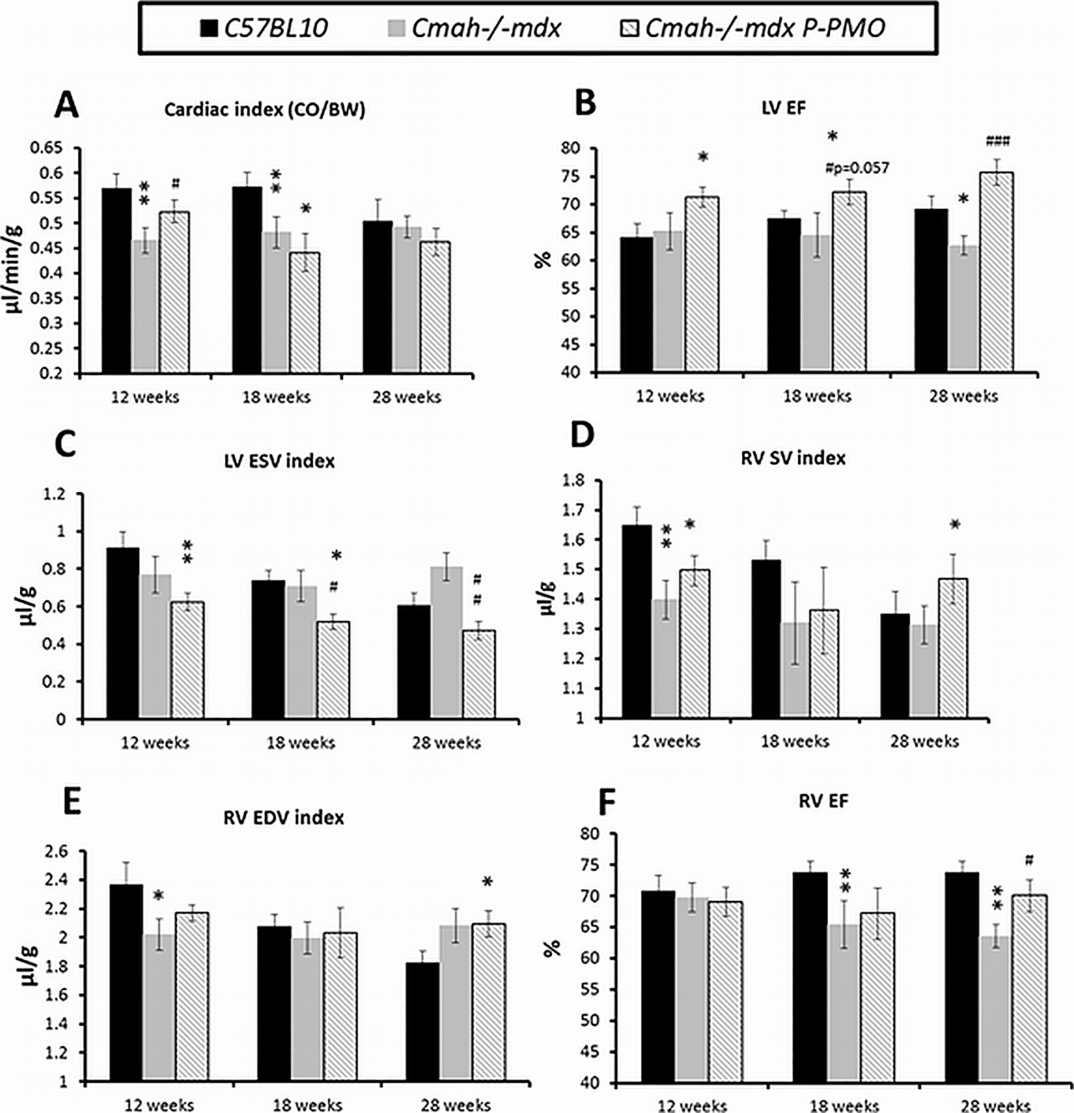
**A-F: P-PMO significantly ameliorates left and right ventricular dysfunction in *Cmah-/-mdx* mice.** Longitudinal MRI data showing A) cardiac index, B) LV ejection fraction and C) LV ESV index. D) RV stroke volume index, E) RV end diastolic volume index and F) RV ejection fraction index (n = 8-13/group) (*p<0.05, **p<0.01 different from *C57BL10*, #p<0.05, ##p<0.01, ###p<0.001 different from *Cmah-/-mdx*, ^p<0.05 different from *mdx*,1-way ANOVA, post-hoc Bonferroni). For clarity of presentation, significant differences between treated *Cmah-/-mdx* and *mdx* controls are not annotated. Error bars represent SEM.

*Cmah-/-mdx* mice show evidence of LV dysfunction as early as 12 weeks with reduced CO (data not shown) and CO to body weight (CO/BW) ratio (Fig 4A). P-PMO treatment delays this reduction in cardiac output however treated animals have reduced CO by 18 weeks (Fig 4A). Interestingly, CO/BW ratio is normal in both the *mdx* and *Cmah-/-mdx* mice by 28 weeks, when *C57Bl10* mice show an age-related reduction in CO/BW ratio. LV EF is significantly reduced in *Cmah-/-mdx* mice at 28 weeks (Fig 4B) and this is prevented by P-PMO treatment by virtue of a marked reduction in LV ESV (Fig 4C).

### P-PMO ameliorates RV dysfunction in *Cmah-/-mdx* mice

*Cmah-/-mdx* mice also show evidence of early RV dysfunction at 12 weeks with a significant reduction in RV SV, RV SV index (Fig 4D) and RV EDV index (Fig 4E). This progresses to reduced RV EF at 18 and 28 weeks (Fig 4F). RV EF is significantly improved by treatment by 28 weeks (Fig 4F).

### P-PMO has direct myocardial effects on cardiac function as well as indirect vascular effects

**Fig 5:**
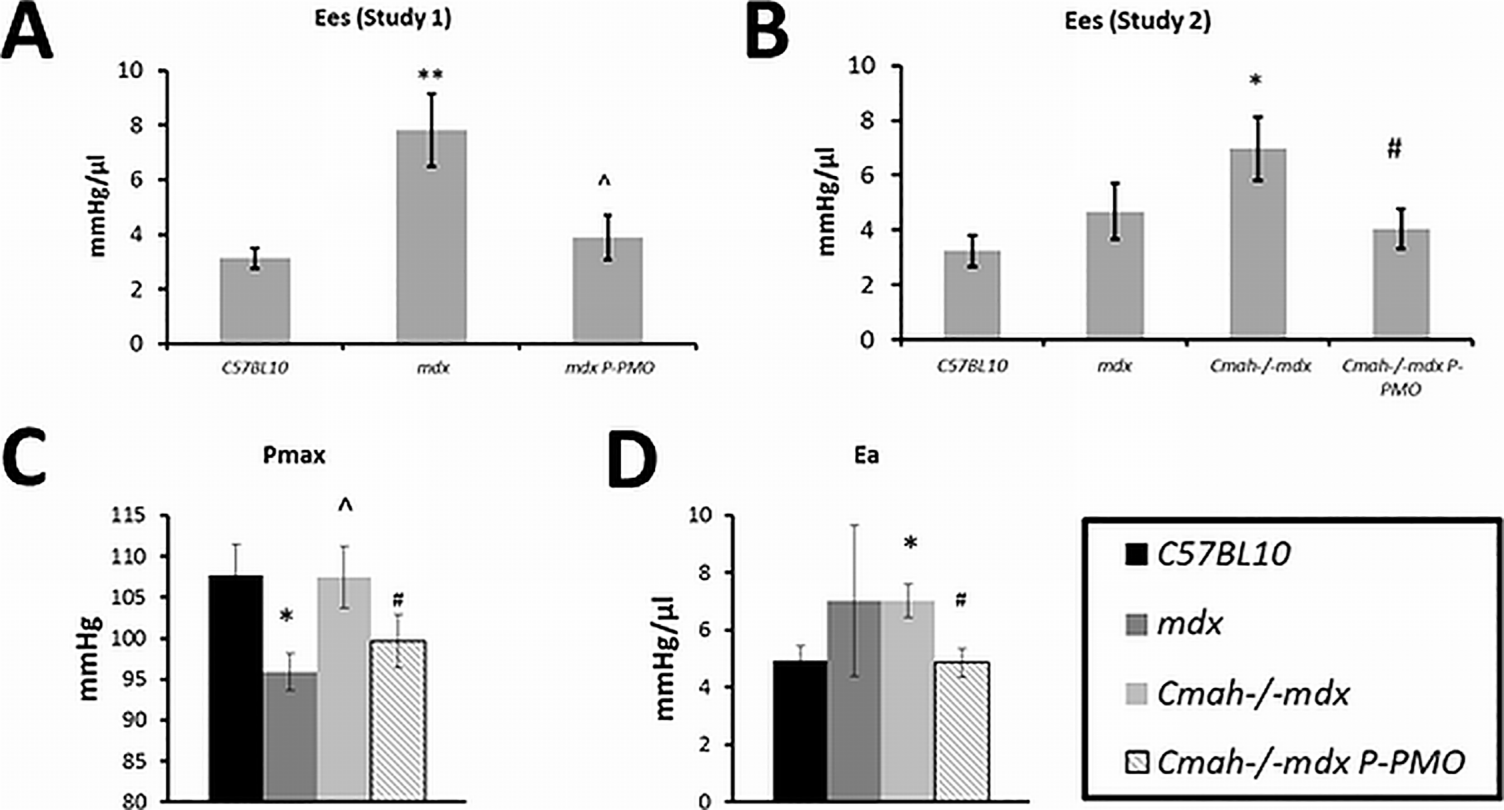
**A-D: P-PMO has direct effects and indirect vascular effects on the myocardium.** There is evidence of a direct myocardial effect of P-PMO in the form of normalised end systolic elastance in both A) Study 1 and B) Study 2. P-PMO also has an indirect effect on the heart through reduction in afterload, decreasing C) maximum pressure and D) arterial elastance. Increased Ees in the DMD models is likely due to systolic stiffening related to fibrosis as we have seen previously26. *p<0.05, **p<0.01 significantly different from *C57BL10*, ^p<0.05 significantly different from mdx, #p<0.05 significantly different from *Cmah-/-mdx*, 1-way ANOVA, post-hoc Bonferroni) (n = 7-8/group). Error bars represent SEM.

Conductance catheter data are highly informative of the mode of action of P-PMO in the heart. There is evidence of direct myocardial effects of P-PMO which can be seen in the normalisation of the load independent measure of contractility, end systolic elastance (E_es)_ in both Study 1 and 2 (Fig 5A & 5B). E_es_ is significantly increased in 32 week old *mdx* mice at baseline and P-PMO acted to reduce this to *C57BL10* levels (Fig 5A). E_es_ was also significantly elevated in 28 week old *Cmah-/-mdx* mice and was reduced to *C57BL10* levels by P-PMO (Fig 5B). End systolic elastance is measured during transient *vena caval* occlusion.

Evidence of indirect effects of P-PMO can be seen with reduced maximum pressure (Fig 5C) and reduced arterial elastance (Ea) (Fig 5D) which is indicative of an effect of P-PMO on the peripheral vasculature.

### *Cmah-/-mdx* respond poorly to dobutamine stress and this is corrected by P-PMO treatment

**Fig 6:**
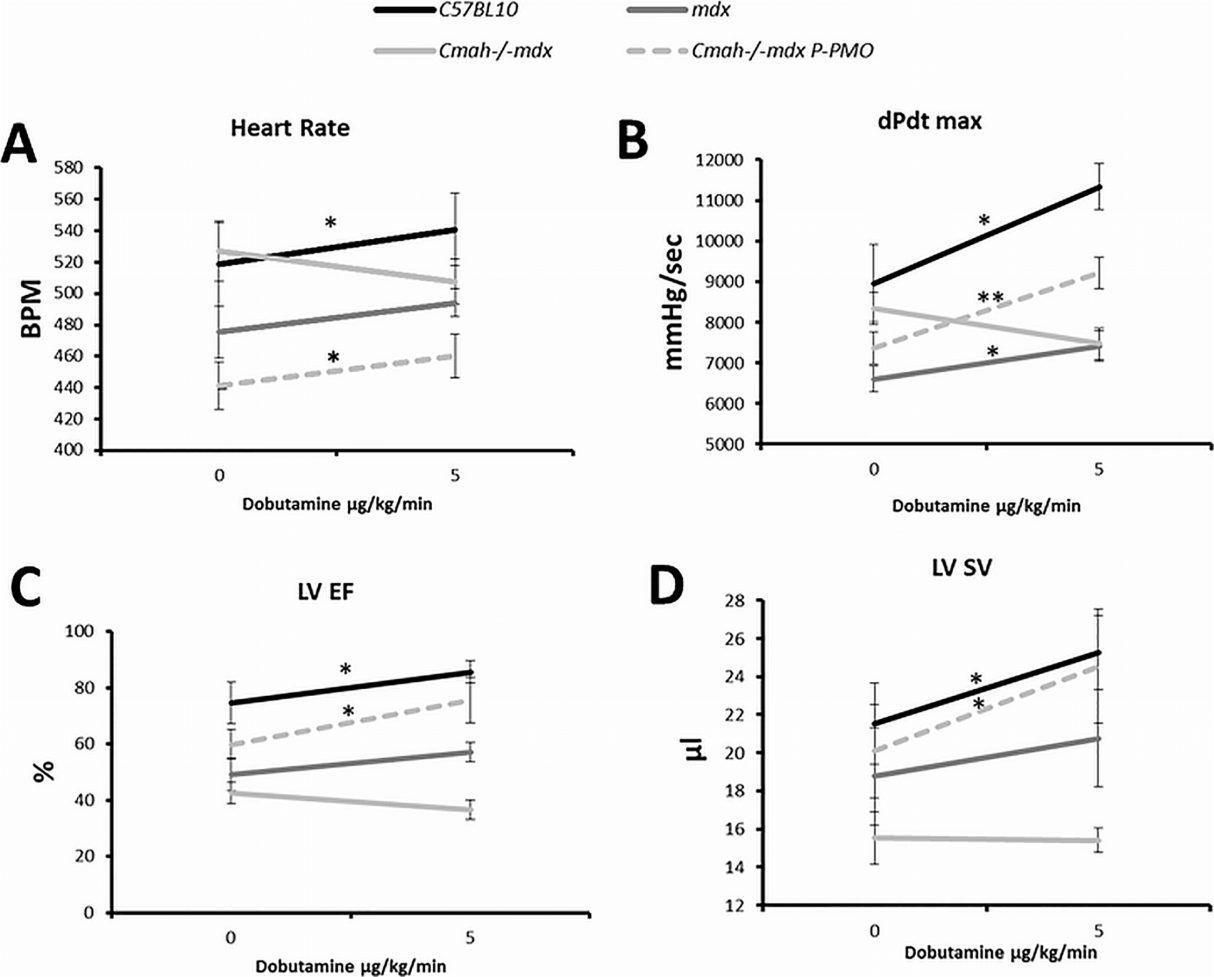
**A-D: P-PMO corrects abnormal dobutamine stress response of *Cmah-/-mdx* mice.** C57BL10 mice demonstrate a normal response to dobutamine stress with elevation of A) Heart rate, B) dPdt max, C) LV EF and D) LV SV. In Cmah-/-mdx these parameters show a blunted response to dobutamine which is corrected by P-PMO treatment. (*p<0.05, significantly different compared to baseline, Paired Student’s t-test) (n = 7-8/group). Error bars represent SEM.

*Cmah-/-mdx* mice show an impaired response to dobutamine stress, failing to increase heart rate, dPdt max, LV EF and LV SV (Fig 6A–6D). *mdx* mice also show a blunted response to dobutamine although to a lesser extent than *Cmah-/-mdx* mice and they show a significant increase in dPdt max. Treatment with P-PMO completely abolishes the abnormal response of *Cmah-/-mdx* mice to dobutamine stress (Fig 6A–6D).

### Effect of treatment on manganese enhancement

**Fig 7:**
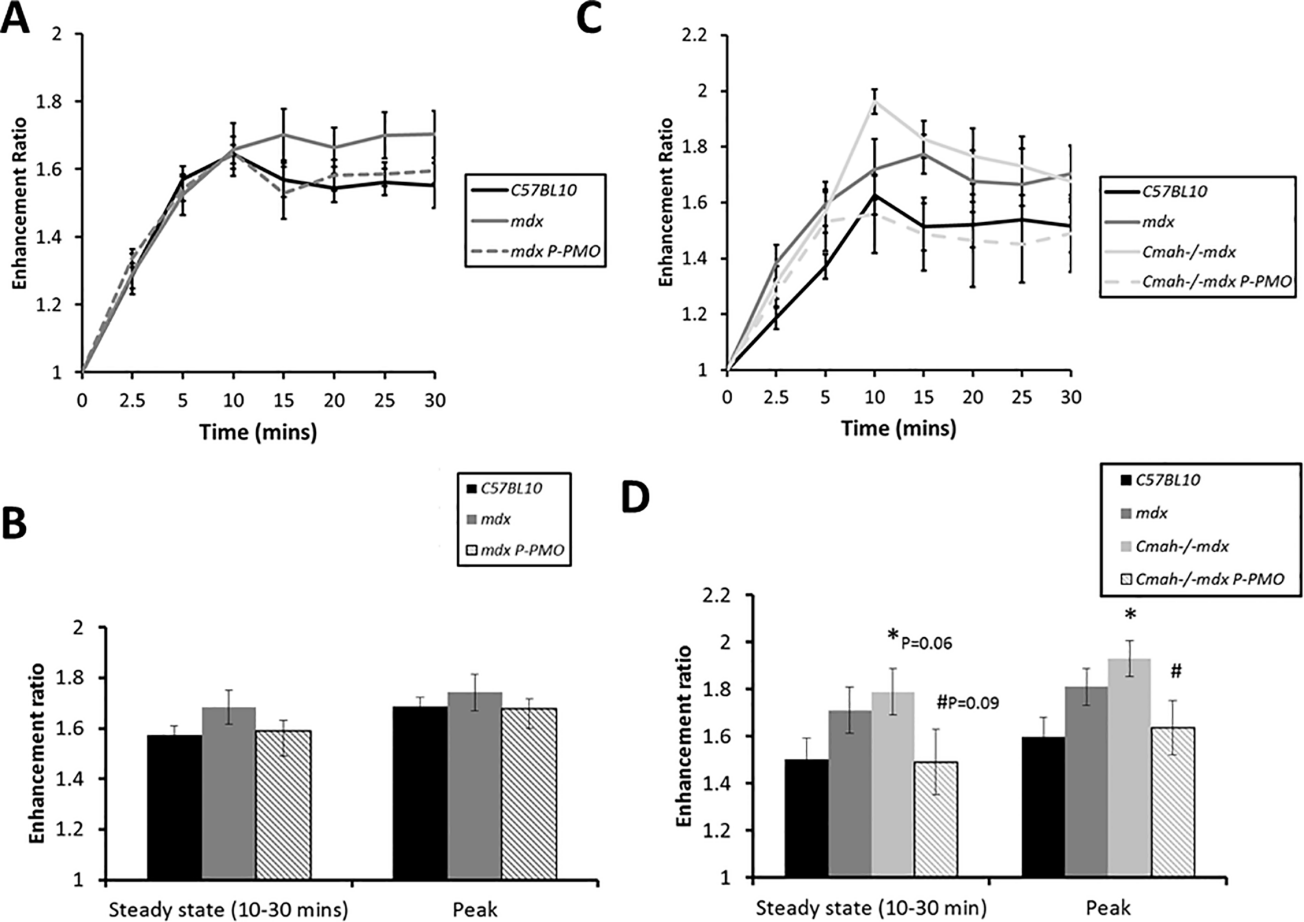
**A-D: P-PMO reduces myocardial manganese enhancement in both *mdx* and *Cmah-/-mdx* mice**. A) manganese enhancement over 30 minutes and B) steady state (10–30 mins)/peak manganese enhancement in Study 1 mdx mice (n = 9-10/group), C) manganese enhancement over 30 minutes and D) Steady state (10–30 mins)/peak manganese enhancement in Study 2 *Cmah-/-mdx* mice (n = 8-13/group) (*p<0.05 different from *C57BL10*, #p<0.05 different from *Cmah-/-mdx*, Unpaired Student’s t-test). Error bars represent SEM.

We have previously shown that manganese (a surrogate marker for myocardial calcium influx) is elevated in *mdx* mice at 24 and 40 weeks of age [20]. In the 32 week old mice we again see a trend towards increased manganese uptake in *mdx* mice (Fig 7A & 7B) although this does not reach significance. P-PMO acted to reduce this aberrant manganese uptake (Fig 7B). Like *mdx m*ice, 28 week old *Cmah-/-mdx* mice showed elevated manganese uptake at baseline (Fig 7C) and peak manganese uptake was significantly elevated from control (Fig 7D). This was similarly corrected by P-PMO treatment (Fig 7D) and peak manganese uptake was significantly reduced in P-PMO treated animals. It should however be noted that although heart rates during MRI in the first study were not significantly different between mice there was a significant reduction in heart rate in P-PMO treated *Cmah-/-mdx* mice in Study 2 which may partially explain the reduction in manganese enhancement in these animals.

## Discussion

### P-PMO treatment restores cardiac dystrophin and has benefits for cardiac function

Our study shows that 8–10 fortnightly IV injections of a moderately high dose of Pip6a-PMO are sufficient to bring about an excellent restoration of dystrophin in TA and diaphragm as well as a functionally significant restoration in heart. The level of restoration in diaphragm and heart was very similar to the levels which have been reported before for a single 12.5 mg/kg IV injection which may suggest that dystrophin restoration is not cumulative with successive injections, at this injection interval and in these tissues [16]. Dystrophin restoration was however much higher in the TA than has been previously reported in another leg muscle, the quadriceps [16]. *Mdx* had a compensated cardiomyopathy at baseline with preserved ejection fraction however Pip6a-PMO was efficacious in its ability to normalise cardiac output and heart rate. *Cmah-/-mdx* mice had significant left ventricular and right ventricular dysfunction at 12 weeks, both of which were corrected by P-PMO treatment.

These data suggest that cardiac dystrophin restoration of 37 and 52% (as measured on immunofluorescent staining) in the treated *mdx* and *Cmah-/-mdx* mice respectively, is sufficient to bring significant functional benefit. This is in line with previous studies which showed functional benefit in muscle at 20% dystrophin restoration [23]. Previous studies in heart have suggested that functional benefit can be seen with a much lower dystrophin restoration of 2% [24]. There may therefore be some scope to reduce dose/frequency of dosing in order minimise side effects and to avoid the apparent cumulative toxicity seen in this study at the initial dose of 18 mg/kg.

Recent biodistribution data for Pip6a-PMO suggest that it accumulates to high levels in the kidney and liver [18]. It would therefore be important that the structure of the peptide conjugate is carefully engineered to minimise uptake into these organs and that kidney and liver accumulation is closely monitored. It would also be important to characterise the brown staining cells/aggregates observed in the hearts of treated animals. Melanin-rich cells such as melanomacrophage and melanin aggregates are known to stain brown on H&E and have been used as a biomarker for toxicity in other species such as fish [25]. It is therefore vital to ascertain if the histological changes observed in treated mice are an indicator of toxicity.

### Restoration of dystrophin by P-PMO has consequences for calcium homeostasis

Pip6a-PMO decreased manganese uptake in *Cmah-/-mdx* mice suggesting that restoration of dystrophin using Pip6a-PMO has favourable effects on calcium homeostasis. This is a very encouraging result that suggests the restoration of dystrophin has real functional consequences for membrane stability and/or calcium handling protein function/expression. Furthermore, the restoration of γ-sarcoglycan and nNOS at the muscle membrane in treated animals suggests that dystrophin restoration by Pip6a-PMO may be sufficient to restore other DGC complex proteins and nNOS tethering to the membrane.

### Early P-PMO treatment prevents cardiac remodelling

*Pip6a-PMO* did not however have any obvious positive effect on cardiac pathology when the treatment was initiated at 18 weeks and continued until 32 weeks. Mismatch between pathology and function has been noted in a number of cardiac studies in mice and highlights the importance of examining *in vivo* function as well as histological features *post mortem*. Where the treatment was initiated earlier in the *Cmah-/-mdx* mice (8 weeks) and continued until 28 weeks there was a reduction in myocardial fibrosis. This may suggest a window of opportunity to prevent right ventricular dysfunction which is an early cardiac manifestation evident from as early as 12 weeks in the *Cmah-/-mdx* mice. Early diagnosis and treatment of DMD patients may therefore be key to the success of exon skipping therapies in the future, with increased potential to reduce cardiac remodelling and the associated replacement of healthy myocardium with fibrosis.

### P-PMO treated animals respond more favourably to dobutamine stress and have increased contractile reserve

*mdx* and *Cmah-/-mdx* mice exhibited minimal differences in cardiac function at 28 weeks under baseline conditions. However, *Cmah-/-mice* performed significantly more poorly under dobutamine stress, suggesting reduced adrenergic myocardial contractile reserve in these animals. Control of cardiac contractility is mediated by the ability of a cardiomyocyte to regulate intracellular calcium through stimulation of β-adrenergic receptors (β-ARs), which are known to have changed expression and/or activity in heart failure [26]. The ability of P-PMO to almost completely ameliorate the abnormal response to dobutamine in *Cmah-/-mdx* mice is encouraging and suggests that P-PMO has potential to increase contractile reserve, presumably though direct or indirect effects on β-AR expression and/or sensitivity, and potentially prevent deterioration in cardiac function. This is supported by the reduction in fibrosis seen histologically.

### P-PMO has direct myocardial effects as well as indirect effects on the vasculature

Data from this study are the first to demonstrate the mode of action of P-PMO in the heart. Evidence from conductance catheter suggests that P-PMO has a direct myocardial effect but also an effect on afterload. Somewhat unexpectedly, our 32 week old *mdx* mice and 28 week old *Cmah-/-mdx* had increased end systolic elastance (E_es_) at baseline (we have previously shown decreased E_es_ in younger *mdx* mice [27]. E_es_ has however been shown to be increased in patients with heart failure but preserved ejection fraction, and can be indicative of ventricular systolic stiffness [28]. Indeed, in our previous study of steroids in Delta-sarcoglycan null mice, E_es_ was clearly increased in hearts with more severe fibrosis, highlighting the importance of interpreting E_es_ in the context of other functional parameters [29]. Very strikingly, in the current study, Pip6a-PMO reduced E_es_ to baseline levels which may suggest a very positive effect of treatment on left ventricular compliance.

P-PMO also appears to have indirect effects on the vasculature, reducing maximum pressure and arterial elastance in *Cmah-/-mdx* mice. These data may therefore suggest that dystrophin restoration in the myocardium may have consequences for vascular tone, perhaps due to changes in tethering of nNOS to the sarcolemma. Alternatively, these indirect myocardial effects may be mediated by dystrophin restoration in vascular smooth muscle and the associated restoration of NO-dependent modulation of α-adrenergic vasoconstriction.

## Summary

Overall, our study suggests a very positive effect of P-PMO treatment on cardiac function in two different mouse models of DMD. Given the gravity of cardiomyopathy in DMD, these are encouraging findings. However, much work is still to be done to optimise the structure of the P-PMO in order to achieve clinically relevant improvements in function with minimal toxicity.

## Supporting information

S1 Table Body mass of mice at the end of study 1 (32 weeks). (PDF)

S2 Table Body mass of mice at end of study 2 (28 weeks) (*P<0.05 compared to *C57BL10*). (PDF)

S3 Table Heart mass at end of study 2 (28 weeks). (PDF)

S1 Fig Timelines outlining the protocol followed for the two studies. (PDF)

S2 Fig Relationship between exon skip and dystrophin restoration. A) Study 1 exon skip (RT-PCR) in Pip6A-PMO treated *mdx* mice. B) Significant correlation of exon skip with dystrophin restoration (quantified by IF), (Pearson Correlation (1-sided), p = 0.0142).
(PDF)

S3 Fig P-PMO treatment restores dystrophin to the tibialis anterior (TA) and diaphragm (DIA). A) IF staining of dystrophin in TA and DIA. Quantification of dystrophin restoration on dystrophin/laminin IF in B) TA (*C57BL10* n = 3, *mdx* n = 6, *mdx* Pip6a-PMO n = 8) and C) DIA (*C57BL10* n = 2, *mdx* = 5, *mdx* Pip6a-PMO = 8). Average dystrophin restoration was 96.35% and 47.02%, respectively (***p<0.001 and *p<0.05).
(PDF)

S4 Fig Effect of P-PMO treatment on pathology in tibialis anterior and diaphragm. P-PMO has no significant effect on prevalence of A) centralised nuclei (quantified on H&E) (n = 5/group) but B-C) reduces fibre size heterogeneity and increases average fibre diameter in TA (quantified on laminin IF). (*compared to *C57BL10*, ^^^p<0.001 compared to untreated *mdx*). (*C57BL10* n = 2, *mdx* and *mdx* Pip6a-PMO n = 6/group). C) H&E section demonstrating a reduction in fibre size heterogeneity and reduced numbers of small diameter cells in P-PMO treated *mdx* mice. P-PMO treatment reduces diaphragm fibrosis in *mdx* mice. D) H&E stained transverse and longitudinal sections of diaphragm demonstrating a clear reduction in fibrosis with P-PMO treatment.
(PDF)

S5 Fig Effect of P-PMO treatment on nNOS and γ-Sgcd expression and localisation in *mdx* mice. Scale bar = 100 μm.
(PDF)
