# Supplementary material for: Peptide-conjugated phosphodiamidate oligomer-mediated exon skipping has benefits for cardiac function in mdx and Cmah-/-mdx mouse models of Duchenne muscular dystrophy
Source: PLoS One. 2018 Jun 18;13(6):e0198897. doi: 10.1371/journal.pone.0198897 (PMC6005479; doi:10.1371/journal.pone.0198897)
Supplement: S1 Table — (PDF) [file pone.0198897.s001.pdf]

S1 Table: Body mass of mice at the end of study 1 (32 weeks).

	**End body mass (g) (±S.E.M)**
C57BL10	36.8 (± 0.7)
mdx	36.6 (± 0.3)
mdx Pip6a-PMO	35.7 (± 1.0)
