# Supplementary material for: Peptide-conjugated phosphodiamidate oligomer-mediated exon skipping has benefits for cardiac function in mdx and Cmah-/-mdx mouse models of Duchenne muscular dystrophy
Source: PLoS One. 2018 Jun 18;13(6):e0198897. doi: 10.1371/journal.pone.0198897 (PMC6005479; doi:10.1371/journal.pone.0198897)
Supplement: S2 Table — (PDF) [file pone.0198897.s002.pdf]

S2 Table: Body mass of mice at end of study 2 (28 weeks) (*P<0.05 compared to *C57BL10*).

	**End body mass (g) (±S.E.M)**
C57BL10	38.6 (±1.2)
mdx	35.6 (± 0.8)
Cmah-/-mdx	33.7 (±1.3)*
Cmah-/-mdx Pip6a-PMO	33.4 (±1.3)*
