# Supplementary material for: Peptide-conjugated phosphodiamidate oligomer-mediated exon skipping has benefits for cardiac function in mdx and Cmah-/-mdx mouse models of Duchenne muscular dystrophy
Source: PLoS One. 2018 Jun 18;13(6):e0198897. doi: 10.1371/journal.pone.0198897 (PMC6005479; doi:10.1371/journal.pone.0198897)
Supplement: S3 Table — (PDF) [file pone.0198897.s003.pdf]

S3 Table: Heart mass at end of study 2 (28 weeks)

	**End heart mass (mg) (±S.E.M)**
C57BL10	146.5 (±5.5)
mdx	156.9 (±4.7)
Cmah-/-mdx	154.2 (±9.9)
Cmah-/-mdx Pip6a-PMO	141.1 (±6.8)
