# Supplementary material for: Peptide-conjugated phosphodiamidate oligomer-mediated exon skipping has benefits for cardiac function in mdx and Cmah-/-mdx mouse models of Duchenne muscular dystrophy
Source: PLoS One. 2018 Jun 18;13(6):e0198897. doi: 10.1371/journal.pone.0198897 (PMC6005479; doi:10.1371/journal.pone.0198897)
Supplement: S2 Fig — A) Study 1 exon skip (RT-PCR) in Pip6A-PMO treated mdx mice. B) Significant correlation of exon skip with dystrophin restoration (quantified by IF), (Pearson Correlation (1-sided), p = 0.0142). (PDF) [file pone.0198897.s005.pdf]

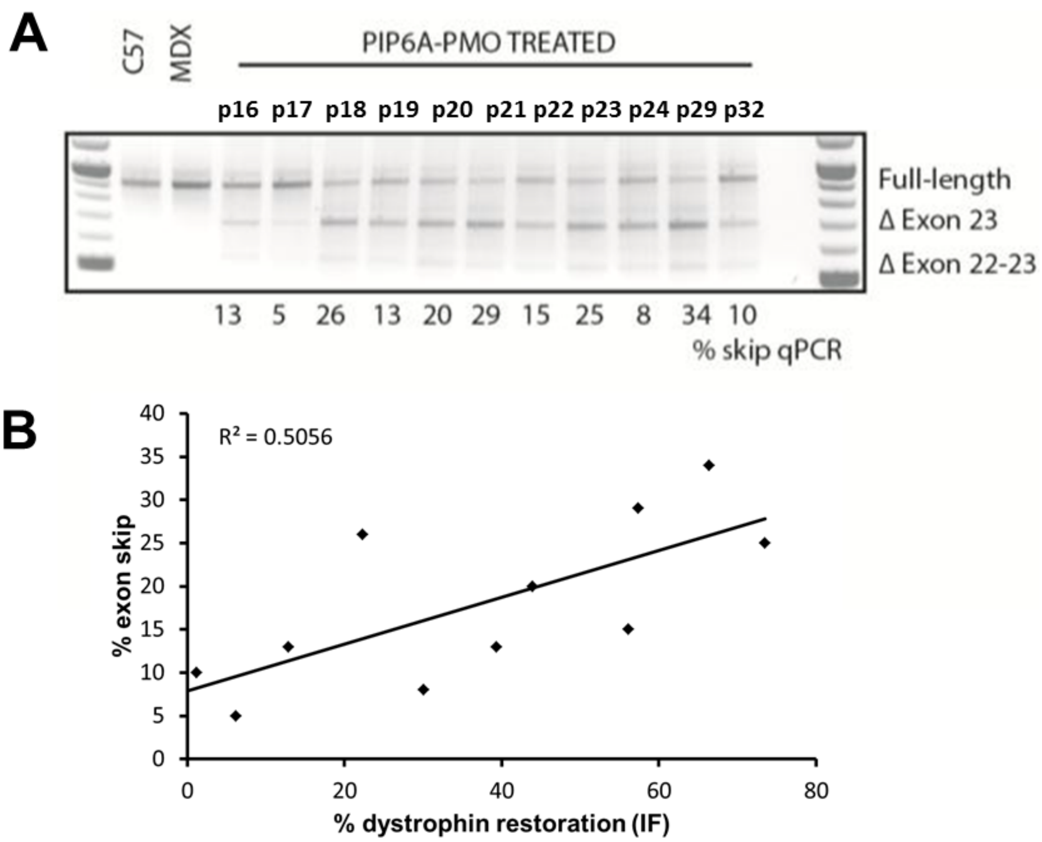

S2 Fig: Relationship between exon skip and dystrophin restoration A) Study 1 exon skip (RT-PCR) in Pip6A-PMO treated *mdx* mice. B) Significant correlation of exon skip with dystrophin restoration (quantified by IF), (Pearson Correlation (1-sided), p=0.0142).
