# Supplementary material for: Peptide-conjugated phosphodiamidate oligomer-mediated exon skipping has benefits for cardiac function in mdx and Cmah-/-mdx mouse models of Duchenne muscular dystrophy
Source: PLoS One. 2018 Jun 18;13(6):e0198897. doi: 10.1371/journal.pone.0198897 (PMC6005479; doi:10.1371/journal.pone.0198897)
Supplement: S3 Fig — A) IF staining of dystrophin in TA and DIA. Quantification of dystrophin restoration on dystrophin/laminin IF in B) TA (C57BL10 n = 3, mdx n = 6, mdx Pip6a-PMO n = 8) and C) DIA (C57BL10 n = 2, mdx = 5, mdx Pip6a-PMO = 8). Average dystrophin restoration was 96.35% and 47.02%, respectively (***p<0.001 and *p<0.05). (PDF) [file pone.0198897.s006.pdf]

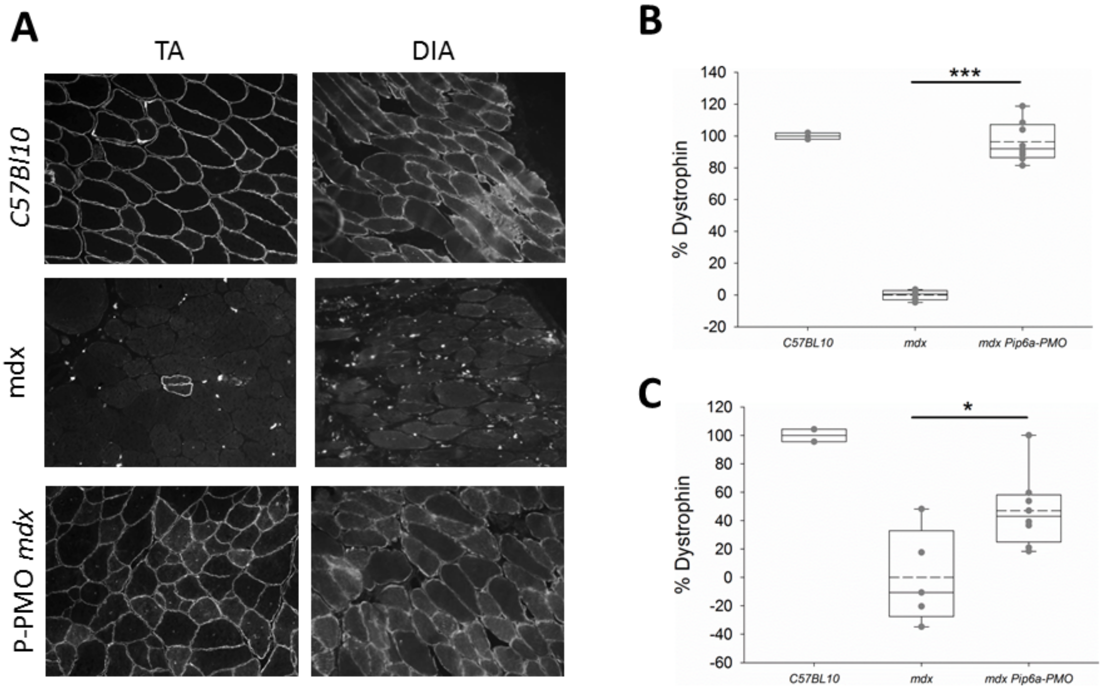

S3 Fig: P-PMO treatment restores dystrophin to the tibialis anterior (TA) and diaphragm (DIA). A) IF staining of dystrophin in TA and DIA. Quantification of dystrophin restoration on dystrophin/laminin IF in B) TA (*C57BL10* n=3, *mdx* n=6, *mdx* Pip6a-PMO n=8) and C) DIA (*C57BL10* n=2, *mdx*= 5, *mdx* Pip6a-PMO=8). Average dystrophin restoration was 96.35% and 47.02%, respectively (***p<0.001 and *p<0.05).
