# Supplementary material for: Peptide-conjugated phosphodiamidate oligomer-mediated exon skipping has benefits for cardiac function in mdx and Cmah-/-mdx mouse models of Duchenne muscular dystrophy
Source: PLoS One. 2018 Jun 18;13(6):e0198897. doi: 10.1371/journal.pone.0198897 (PMC6005479; doi:10.1371/journal.pone.0198897)
Supplement: S4 Fig — P-PMO has no significant effect on prevalence of A) centralised nuclei (quantified on H&E) (n = 5/group) but B-C) reduces fibre size heterogeneity and increases average fibre diameter in TA (quantified on laminin IF). (*compared to C57BL10, ^^^p<0.001 compared to untreated mdx). (C57BL10 n = 2, mdx and mdx Pip6a-PMO n = 6/group). C) H&E section demonstrating a reduction in fibre size heterogeneity and reduced numbers of small diameter cells in P-PMO treated mdx mice. P-PMO treatment reduces diaphragm fibrosis in mdx mice. D) H&E stained transverse and longitudinal sections of diaphragm demonstrating a clear reduction in fibrosis with P-PMO treatment. (PDF) [file pone.0198897.s007.pdf]

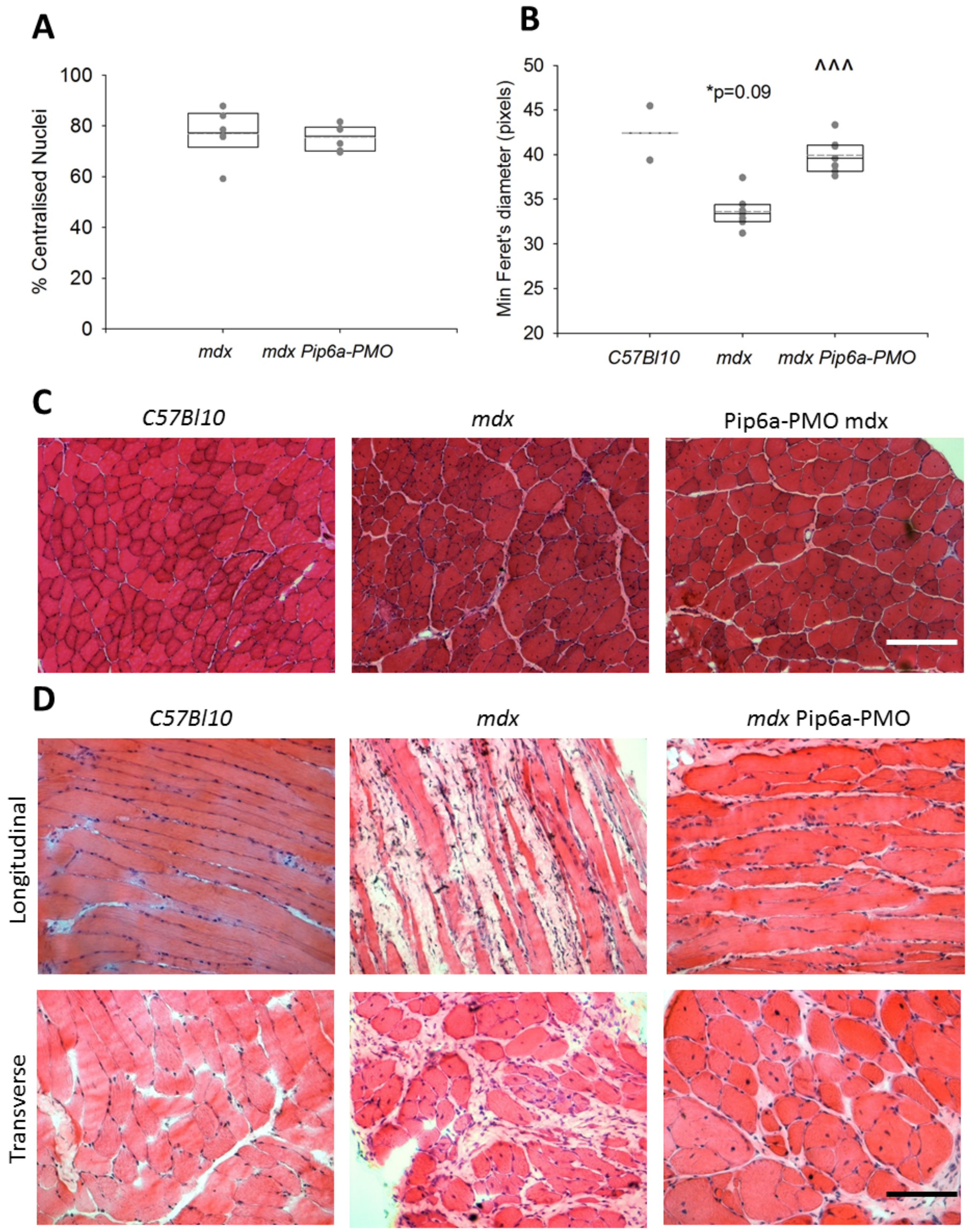

S4 Fig: Effect of P-PMO treatment on pathology in tibialis anterior and diaphragm. P-PMO has no significant effect on prevalence of A) centralised nuclei (quantified on H&E) (n=5/group) but B-C) reduces fibre size heterogeneity and increases average fibre diameter in TA (quantified on laminin IF). (*compared to *C57BL10*, ^^^p<0.001 compared to untreated *mdx*). (*C57BL10* n=2, *mdx* and *mdx* Pip6a-PMO n=6/group). C) H&E section demonstrating a reduction in fibre size heterogeneity and reduced numbers of small diameter cells in P-PMO treated *mdx* mice. P-PMO treatment reduces diaphragm fibrosis in *mdx* mice. D) H&E stained transverse and longitudinal sections of diaphragm demonstrating a clear reduction in fibrosis with P-PMO treatment.
