# Supplementary material for: Peptide-conjugated phosphodiamidate oligomer-mediated exon skipping has benefits for cardiac function in mdx and Cmah-/-mdx mouse models of Duchenne muscular dystrophy
Source: PLoS One. 2018 Jun 18;13(6):e0198897. doi: 10.1371/journal.pone.0198897 (PMC6005479; doi:10.1371/journal.pone.0198897)
Supplement: S5 Fig — Scale bar = 100 μm. (PDF) [file pone.0198897.s008.pdf]

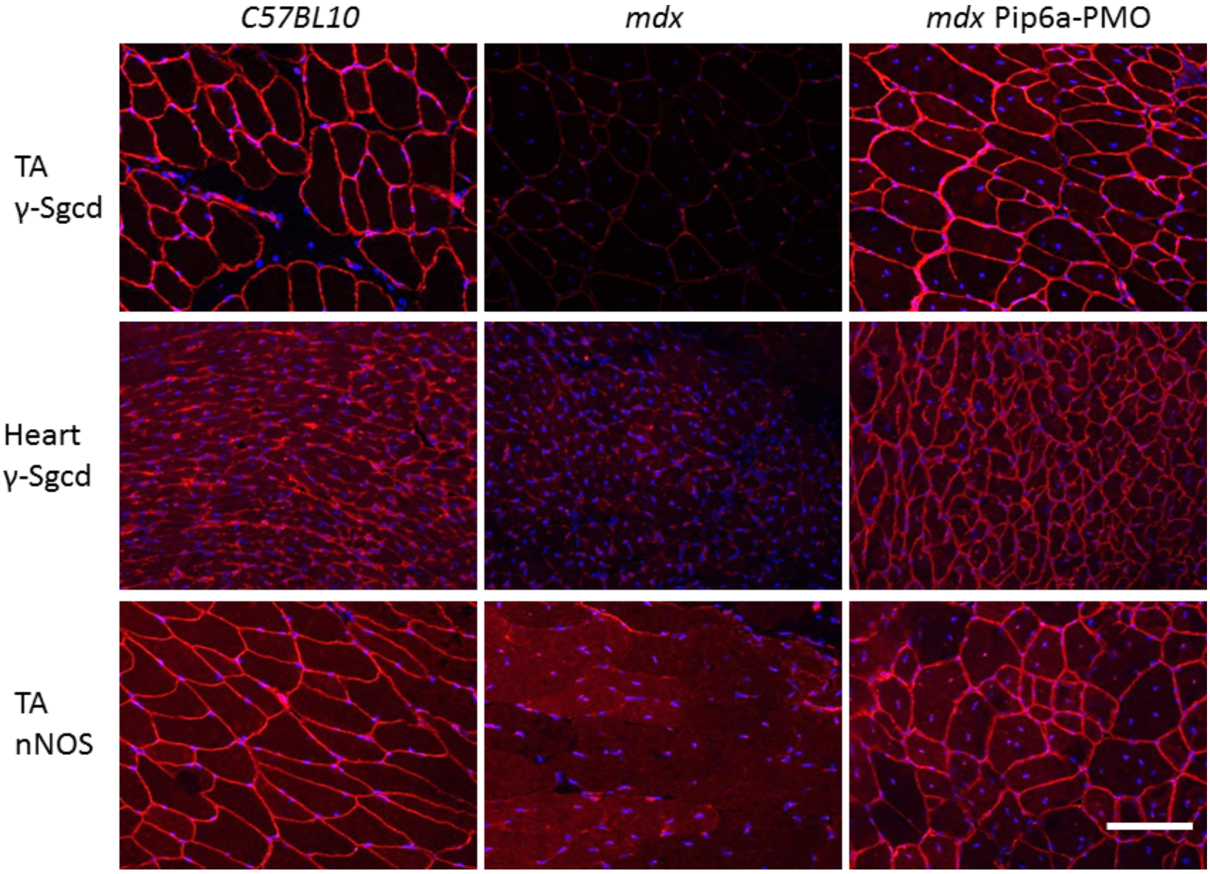

S5 Fig: Effect of P-PMO treatment on nNOS and γ-Sgcd expression and localisation in *mdx* mice. Scale bar =100 μm
